# Splenomegaly, Spleen Amyloidosis and Neutrophil Infiltration are Present in 3xTg-AD, but not Tg-SwDI Mice

**DOI:** 10.1007/s12017-025-08884-8

**Published:** 2025-09-04

**Authors:** Gonzalo Acero, Adrian Rodriguez-Lopez, Georgina Díaz, Daniel Esteban, Mónica Herrera-Ángeles, Goar Gevorkian

**Affiliations:** Instituto de Investigaciones Biomédicas, Universidad Nacional Autónoma de México (UNAM), Cuidad Universitaria, Apartado Postal 70228, CP 04510 CDMX, Mexico

**Keywords:** Alzheimer´s disease, Cerebral amyloid angiopathy, 3xTg-AD mouse, Tg-SwDI mouse, Splenomegaly

## Abstract

It is now widely accepted that the development of neurodegenerative diseases depends on and affects many pathological processes, both in the brain and the periphery. Inflammatory, cardiovascular, metabolic, cerebrovascular, autoimmune, and other environmental factors have been extensively studied and shown to contribute notably to the onset, pathogenesis, and clinical outcome of Alzheimer´s disease (AD), Parkinson´s disease (PD), cerebral amyloid angiopathy (CAA), multiple sclerosis, and other neurological disorders. Likewise, AD-induced changes in other tissues outside the central nervous system, such as abnormalities observed in the liver, spleen, or lungs, have been documented and extensively studied, leading to a better understanding of brain-periphery crosstalk in neurodegenerative diseases and the development of novel diagnostic and therapeutic approaches. In this study, we documented striking differences in the periphery in two frequently used, well-established APP transgenic mouse models of AD: 3xTg-AD mice, harboring three human genes (APP, tau, and Psen1), and Tg-SwDI mice, expressing human APP with the Swedish and vasculotropic Dutch/Iowa mutations in the brain. We documented splenomegaly, immunoglobulin-associated spleen amyloidosis, and an increase in the percentage of neutrophils in the spleen and macrophages in the liver in 3xTg-AD mice but not in age-matched Tg-SwDI mice, which are commonly used as an AD/CAA model. Our data suggest that the results observed in any transgenic mouse strain should be taken into account with caution. A detailed knowledge of pathological characteristics recapitulated in a particular strain can help to determine which mice are more appropriate for studying a specific mechanism or therapeutic approach.

**Supplementary Information:**

The online version contains supplementary material available at 10.1007/s12017-025-08884-8.

## Introduction

It is now widely accepted that the development of neurodegenerative diseases depends on and affects many pathological processes, both in the brain and the periphery. Inflammatory, cardiovascular, metabolic, cerebrovascular, autoimmune, and other environmental factors have been extensively studied and shown to contribute notably to the onset, pathogenesis, and clinical outcome of Alzheimer´s disease (AD), Parkinson´s disease (PD), cerebral amyloid angiopathy (CAA), multiple sclerosis, and other neurological disorders (Gong et al., 2018; Iqbal & Grundke-Iqbal, 2010). Among the changes observed in the brains of AD patients are the accumulation of amyloid beta peptide (Aβ) in the form of extra- and intracellular amyloid aggregates in the parenchyma and the cerebral vasculature, the intracellular accumulation of hyperphosphorylated Tau protein in the form of neurofibrillary tangles (NFTs), neuronal and synaptic loss and damage, in addition to neuroinflammation and oxidative stress (Griffiths & Grant, 2022; Walsh & Selkoe, 2020). Likewise, AD-induced changes in other tissues outside of the central nervous system, such as abnormalities observed in the liver, spleen, or lungs, have been documented and extensively studied, leading to a better understanding of brain-periphery crosstalk in neurodegenerative diseases and the development of novel diagnostic and therapeutic approaches (Lutshumba et al., 2021; Wang et al., 2017).

The most commonly used experimental models for AD studies are human amyloid precursor protein (APP) transgenic mice that exhibit progressive age-related accumulation of intra- and extracellular amyloid aggregates, and, in some cases, intracellular NFTs, as well as brain inflammation, synaptic alterations and cognitive decline (De Plano et al., 2024). Several transgenic strains have been generated and thoroughly characterized to elucidate molecular and cellular processes involved in AD and evaluate numerous therapeutic approaches (De Plano et al., 2024). One of the most applied transgenic mouse models of AD (3xTg-AD mice), harboring three human genes (APP, tau, and Psen1), was generated in 2003 (Oddo et al., 2003). Detailed studies on temporal and regional accumulation of Aβ and NFTs in 3xTg-AD mice have been performed, and many aspects of AD pathogenesis were observed, allowing for optimal design of future experiments in these animals (Belfiore et al., 2019; Mastrangelo & Bowers, 2008). Likewise, 3xTg-AD mice have also been used to elucidate the role of the immune system in the etiology and progression of AD, and alterations in immune cell populations were observed since an early age (Marchese et al., 2014; St-Amour et al., 2019; Nava Catorece et al., 2021). Notably, while female 3xTg-AD mice exhibit more severe amyloid pathology, male mice have a shorter lifespan (Gimenez-Llort et al., 2014). Besides, peripheral immune abnormalities such as a decreased number of T cells, an increased CD3 + CD4-CD8-double-negative (DN) T cells and autoantibody levels, and splenomegaly are more pronounced in males compared with females (Marchese et al., 2014; Kapadia et al., 2018, 2021; St-Amour et al., 2019, Nava Catorece et al., 2021).

Also, a unique transgenic mouse model [Tg-SwDI transgenic mice expressing human Aβ precursor protein (AβPP) harboring the Swedish K670N/M671L and vasculotropic Dutch/Iowa E693Q/D694N mutations in the brain] was developed to investigate the early-onset and robust accumulation of both parenchymal and vascular Aβ and the mechanisms of cerebrovascular dysfunction, neuroinflammation, neurodegeneration, and cognitive decline observed in AD/CAA patients (Davis et al., 2004; Miao et al., 2005; Davis et al., 2006; Xu et al., 2007; Xu et al., 2014; Schiltzova et al., 2017; Robison et al., 2019).

In our previous studies, we documented important immunological abnormalities in the periphery of 3xTg-AD mice and compared the obtained data with known changes in AD patients (Nava Catorce et al., 2021). Here, we investigated age-dependent abnormalities in the spleen and liver in two frequently used mouse models of AD and CAA (3xTg-AD and Tg-SwDI) and found significant differences: age-dependent splenomegaly, spleen amyloidosis, and an increase in the percentage of neutrophils in the spleen and macrophages in the liver were documented in 3xTg-AD mice but not in age-matched Tg-SwDI mice, which are commonly used as an AD/CAA model.

It is worth mentioning that there is not a single transgenic mouse model that precisely replicates all aspects of AD, and several successful preclinical therapeutic interventions in mice have failed in late-stage clinical trials (De plano et al., 2024; Sanchez-Varo et al., 2022). A detailed understanding of the pathological characteristics recapitulated in a particular strain can help to decide which mice are more appropriate for studying a specific mechanism or therapeutic approach.

## Materials and Methods

### Experimental Animals

Experiments were conducted following protocols approved by the Animal Care and Use Committee of our University (Protocol ID: 209) and in compliance with NIH guidelines for the use of experimental animals. Likewise, we adhered, when applicable, to the items of the Arrive 2.0 guidelines (Percie du Sert et al., 2020). Mice were provided with water and a standard diet ad libitum and were maintained on a 12-h light/dark cycle in a temperature-controlled room. Animals with visible injuries due to aggression or other aberrant health conditions were excluded from the study. Control B6129SF2/J or C57BL/6 and homozygous 3xTg-AD (Oddo et al., 2003) and Tg-SwDI (Davis et al., 2004) transgenic mice were maintained by breeding in our facilities. Original mating pairs were obtained from The Jackson Laboratory (Bar Harbor, ME, USA). 3-, 6-, 12-, and 15-month-old 3xTg-AD, 16-month-old Tg-SwDI, and 15–18-month-old control male and female mice were used. To obtain spleen and liver samples, mice were deeply anesthetized with Sevorane (Abbott, Laboratorios Abbott Mexico, Mexico). The spleens were carefully removed, weighed, and photographed. Then the spleens were fixed for 24 h in dPBS (138 mM NaCl/2.7 mM KCl/8.1 mM Na_2_HPO_4_/1.2 mM KH_2_PO_4_) containing 4% formaldehyde, dehydrated in 10%, 20%, and 30% sucrose solution, embedded in Tissue-Plus (Fisher HealthCare, Houston, TX, USA) and sectioned for immunofluorescence analysis using Kedee cryostat (Kedee, Jinhua, Zhejiang, PRC). Another set of isolated spleens was perfused with ice-cold dPBS, erythrocytes were lysed with 2–3 ml of lysing solution (155 mM NH_4_Cl /10 mM KHCO_3_ /96.7 mM EDTA-2Na) for 5 min, and the splenocytes were washed and assessed by flow cytometry. Liver cell suspension for flow cytometry analysis was obtained as described previously (Wyatt-Johnson et al., 2024). Livers were perfused with ice-cold dPBS, carefully dissected, cut into small pieces and transferred through a 70 µm cell strainer into conical tubes using 10 ml of GKN-BSA buffer (0.2% Glucose/0.8% NaCl/ 0.4% KCl/ 0.01 M Na_2_HPO_4_/0.006 M NaH_2_PO_4_/0.02% bovine serum albumin (BSA) (Millipore Corp., Burlington, MA, USA)). The cells were washed by centrifugation, resuspended in GKN-BSA buffer, and the suspension was applied over 4 ml of 40% Percoll solution (Sigma–Aldrich, Saint Louis, MO, USA). After centrifugation at 2000 rpm for 20 min, without brake, at 18 °C, cells were resuspended in dPBS, washed twice with dPBS, and assessed by flow cytometry.

### Immunofluorescence

As described previously, 30 µm-thick spleen sections were processed (Hernandez-Zimbron et al., 2012). Briefly, after antigen retrieval by incubating in citrate buffer (0.01 M citric acid, 0.05% Tween 20, pH 6.0) at 70 °C for 30 min, followed by rinsing in 40% formic acid (Merck, Darmstadt, Germany) for 3 min at room temperature (RT), samples were thoroughly washed several times with TBS, then incubated for 15 min in TBS-0.1% Triton X-100 (TBS-Tx) and blocked with a 2% BSA solution (Millipore Corp., Burlington, MA, USA) in TBS for 20 min at RT. The first set of spleen samples was incubated overnight at 4 °C with rabbit anti-Aβ polyclonal antibodies obtained in our previous work diluted in TBS-Tx containing 5% normal goat serum (VectorLabs, Burlingame, CA, USA). After washing, sections were incubated for 1 h at RT with Alexa Fluor 594 goat anti-rabbit IgG (H + L) (Invitrogen, Eugene, OR, USA). The second set of spleen sections was incubated with Alexa Fluor 594 goat anti-mouse IgG (H + L) (Invitrogen) diluted in TBS-Tx containing 5% normal goat serum (VectorLabs). Thioflavin S (Sigma–Aldrich, St Louis, MO, USA) staining (1% in H_2_OmQ) was performed for 8 min at RT and samples were washed sequentially with 80%, 95%, and 100% ethanol. Finally, spleen sections were mounted on glass slides in Vectashield medium (Vector Laboratories, Burlingame, CA, USA). Samples were examined on a Nikon ECLIPSE Ci microscope equipped with a DP71 camera (Nikon Instruments Inc., Melville, NY, USA). The confocal microscope images were taken on a Fluoview FV10i confocal microscope (Olympus®, Japan) using the 60 × NA 1.35 oil immersion objective (UPLSAPO60XO). ImageJ 1.53e software (NIH, USA) was applied for processing and analyzing images and for 3D reconstruction of confocal images.

### Flow Cytometry

Spleen or liver cells, isolated as described above, were assessed for their live vs dead status using Zombie Aqua™ Fixable Viability Kit (BioLegend, San Diego, CA, USA). Then, cells were washed with FACS Buffer (dPBS containing 5% Fetal Bovine Serum and 0.02% sodium azide), incubated with Purified Anti-Mouse CD16/CD32 (clone 2.4G2, Tonbo, San Diego, CA, USA), and stained with a panel of fluorescence‐labeled antibodies according to the manufacturer’s instructions: PE anti-mouse CD45.2 (BioLegend); APC anti-mouse/human CD11b (clone M1/70, BioLegend); APC anti-mouse Ly6G (clone 1A8, BioLegend); Brilliant Violet 421™ anti-mouse F4/80 (clone BM8, BioLegend); FITC anti-mouse/human CD45R/B220 (clone RA3-6B2, BioLegend); PE-Cyanine5 Anti-Mouse CD3e (clone 145-2C11, Tonbo). After washing, cells were acquired on an Attune NxT Flow Cytometer (Thermo Fisher Scientific, Waltham, MA), and the results were analyzed using FlowJo 10.10 software (BD, San Jose, CA, USA); at least 20,000 total events were collected.

### Statistical Analysis

Data were analyzed by one-way parametric ANOVA. Dunnett’s test was used to adjust for multiple comparisons. Differences were considered statistically significant when P < 0.05* or < 0.01** or < 0.001***. All analyses were performed using the GraphPad Prism 9.5 software package (GraphPad Software Inc., San Diego, CA, USA). All data are expressed as mean ± standard deviation (SD).

## Results

### Spleens were Enlarged in 3xTg-AD but not Tg-SwDI Mice

**Fig. 1:**
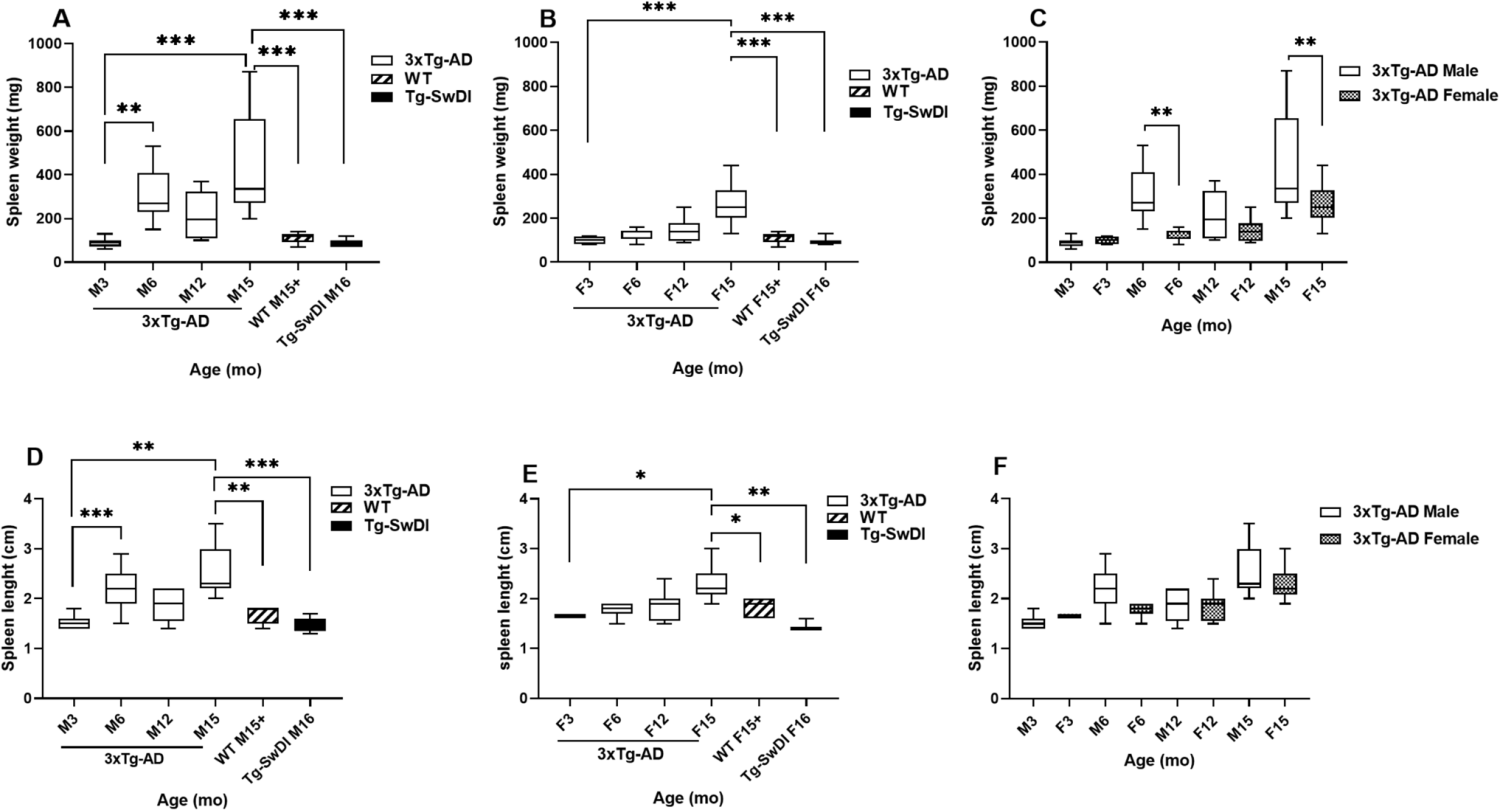
Age-dependent enlargement of the spleen in male and female 3xTg-AD mice. The spleen weight of 3-, 6-, 12-, and 15-month-old male **A** and female **B** 3xTg-AD mice. **C** Comparison of the spleen weight in male and female 3xTg-AD mice. **D** The spleen length of 3-, 6-, 12-, and 15-month-old male **D**) and female **E**) 3xTg-AD mice. No splenomegaly was observed in 16-month-old Tg-SwDI mice or 15–18-month-old non-transgenic animals. **F**) Comparison of the spleen length in male and female 3xTg-AD mice. No splenomegaly was observed in 16-month-old Tg-SwDI mice or 15–18-month-old non-transgenic animals. *p < 0.05, **p < 0.01, ***p < 0.001

In male 3xTg-AD mice, the spleen enlargement was already observed at 6 months of age (weight: 315 ± 127 mg; length: 2.19 ± 0.4 cm; *n* = 11), and the spleens further increased with age, reaching 426 ± 205 mg in 15-month-old animals (Fig. 1). In contrast, in female 3xTg-AD mice, statistically significant differences compared with young animals were observed in 15-month-old animals (weight: 266 ± 88 mg; length: 2.27 ± 0.3 cm; *n* = 14) (Fig. 1). Spleen enlargement was more pronounced in 6- and 15-month-old 3xTg-AD males than in females. Neither control male (weight: 71 ± 15 mg; length: 1.67 ± 0.17 cm; *n* = 7) or female (weight: 111 ± 24 mg; length: .83 ± 0.17 cm; *n* = 7) nor Tg-SwDI transgenic male (weight: 84 ± 15 mg; length: 1.46 ± 0.13 cm; *n* = 11) or female (weight: 100 ± 26 mg; length: 1.47 ± 0.11 cm; *n* = 5) mice had enlarged spleens at 15–18 months of age (Fig. 1).

### Spleen Amyloidosis in 3xTg-AD Mice

**Fig. 2:**
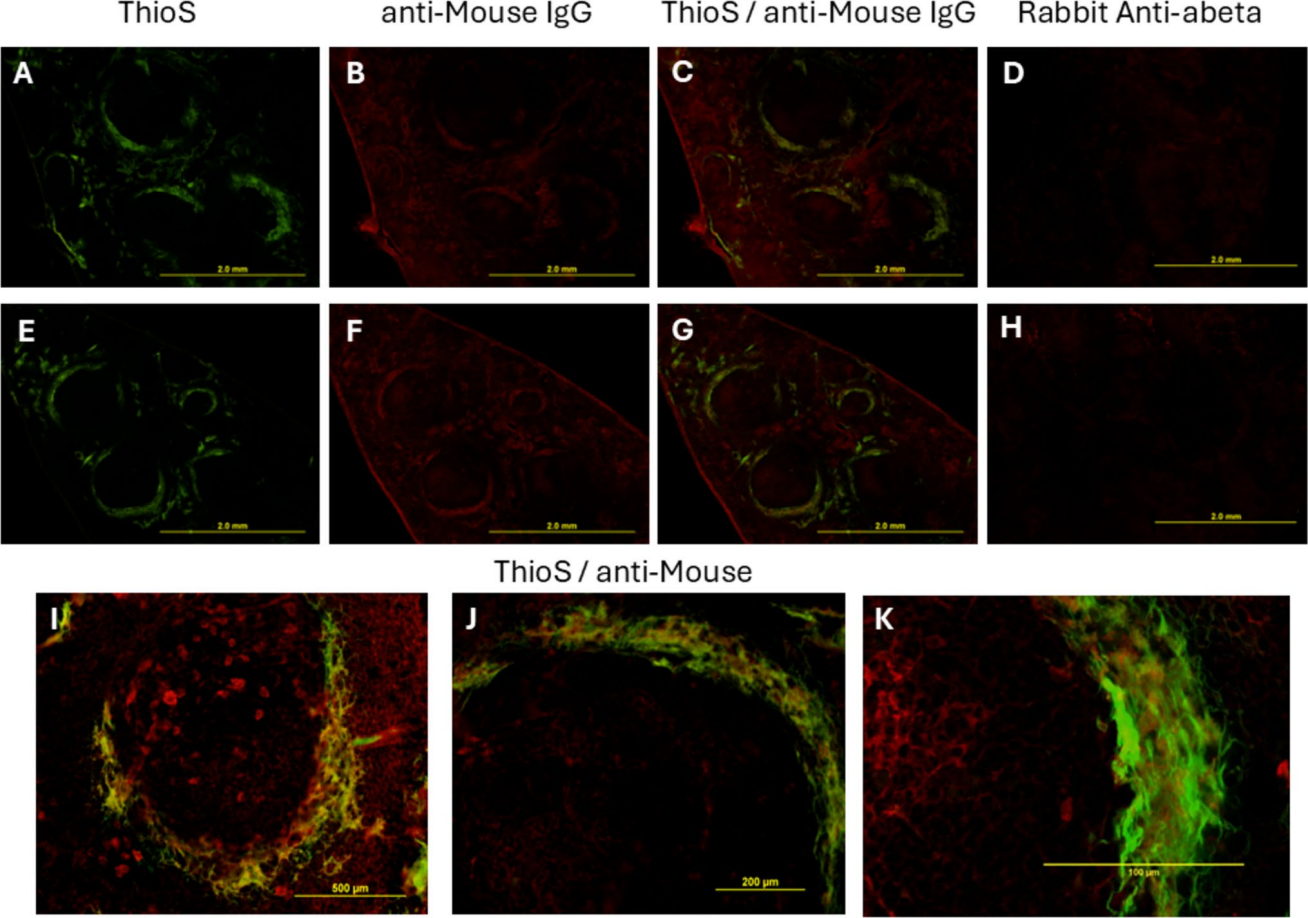
Amyloidosis in the spleen of 3xTg-AD mice. The spleens were stained with ThioS, anti-mouse IgG or anti-Aβ antibodies as described in Materials and Methods. **A** and **E**: representative images of ThioS-positive amyloid; **B** and **F** mouse immunoglobulin-positive areas; C: merge between green (**A**) and red (**B**) channels; **G** merge between green (**E**) and red (**F**) channels; **D** and **H**: no staining with anti-Aβ antibodies is observed. **I**, **J** and **K**: confocal microscopy images showing ThioS- and mouse IgG-positive areas. **A**–**H**: scale bars = 2 mm; **I**: scale bar = 500 µm; **J**: scale bar = 200 µm; **K**: scale bar = 100 µm

To determine amyloid accumulation in the spleen, staining with Thioflavin S was performed. Spleens from 3xTg-AD mice had green ThioS-stained areas (Fig. 2A and E), while no staining was observed in the spleens from Tg-SwDI and control non-transgenic animals (data not shown). To further characterize amyloid aggregates present in the spleen from 3xTg-AD mice, anti-Aβ antibodies were applied, and no staining was observed (Fig. 2D and H). These results suggest that the spleen amyloidosis in 3xTg-AD mice is not caused by Aβ peptide. Yet, mouse IgG-positive deposits (Fig. 2B and F) that were also stained with ThioS were detected (Fig. 2C, G, J–K), pointing to immunoglobulin-associated spleen amyloidosis.

### Neutrophil Infiltration in the Spleen of 3xTg-AD Mice

**Fig. 3:**
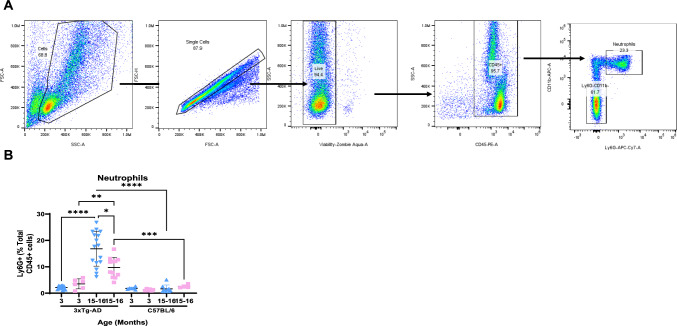
Neutrophil infiltration in the spleen of 3xTg-AD mice. **A** The gating strategy used for flow cytometry analysis of the splenocytes. After forward versus side scatter (FSC vs SSC) gating, single cells were selected and gating for live cells was performed (negative for Aqua Zombie). Then, CD45+ cells were selected and split into Ly6G+ (neutrophils) and Ly6G− cells. **B** Comparison of neutrophil content, expressed as a percentage of CD45+ cells, in the spleen of 3- and 15–16-month-old male (blue) and female (light rose) 3xTg-AD and wild-type mice

To determine whether splenomegaly led to neutrophil infiltration into the spleen, we performed a flow cytometry analysis of the spleen cells from 3xTg-AD mice. The gating strategy and representative dot plots are shown in Fig. 3A. Quantitative analysis revealed an increase in the percentage of neutrophils (Ly6G + cells) among viable CD45 + positive cells in the spleen in 15–16-month-old male (*n* = 16) and female (*n* = 9) 3xTg-AD mice compared with age-matched controls (males *n* = 12; females *n* = 4) (Fig. 3B). Likewise, age-dependent increase of neutrophils in old 3xTg-AD mice compared with young animals (3-month-old males (*n* = 12) and females (*n* = 6)) was documented, while no differences were observed in young and old control C57BL/6 J mice (Fig. 3B). Neutrophil infiltration was higher in male mice compared with females. No increase in the percentage of neutrophils was detected in the spleen of Tg-SwDI mice.

### Increased Macrophage Number in the Liver of 3xTg-AD Mice

**Fig. 4:**
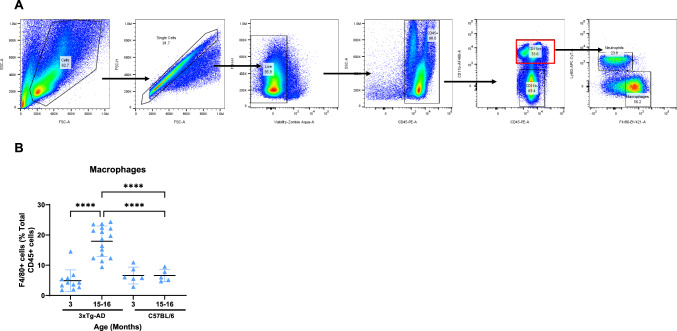
Increased macrophage number in the liver of 3xTg-AD mice. **A** The gating strategy used for flow cytometry analysis of the liver cells. After forward versus side scatter (FSC vs SSC) gating, single cells were selected and gating for live cells was performed (negative for Aqua Zombie). Then, CD45+ cells were selected and split into CD11b+ and CD11− cells. Subsequently, CD11+ cells were selected and split into F4/80+ (macrophages) and F4/80− cells. **B** Comparison of macrophage content, expressed as a percentage of CD45+ cells, in the liver of 3- and 15–16-month-old male 3xTg-AD and wild-type mice

To further explore whether liver inflammation is manifested in 3xTg-AD mice, we performed a flow cytometry analysis of the liver cells from 3xTg-AD mice. The gating strategy and representative dot plots are shown in Fig. 4A. We documented a significant increase of F4/80-positive cells in 15–16-month-old 3xTg-AD males (*n* = 16) compared with 3-month-old mice (*n* = 11) (Fig. 4B) but not females (data not shown). No increase in F4/80-positive cells was observed in 15–16-month-old Tg-SwDI or C57BL/6 J mice (*n* = 5).

## Discussion

In the present study, we documented splenomegaly at 6 months of age in male and 15 months of age in female 3xTg-AD mice, the commonly used transgenic mouse strain for AD studies and therapeutics assessment. Splenomegaly became more severe with age and was more pronounced in males than in females. Our results in old (12 or more months) male and female mice are consistent with previous reports in 3xTg-AD mice; in addition, in this study, we documented for the first time the splenomegaly in 6-month-old male animals (Baeta-Corral et al., 2018, 2023; Barber et al., 2024; Fraile-Ramos et al., 2024; Kapadia et al., 2018; Magistri et al., 2016; Manna et al., 2021; Marchese et al., 2014; Yang et al., 2015). First, the possible cause of the splenomegaly observed in 3xTg-AD mice was hypothesized to be the expression of human APP and presenilin 1 (PS1) with familial AD mutations; still, the results in other APP/PS1 transgenic mice refuted this hypothesis (Manna et al., 2021). Thus, in 18- and 24-month-old 5xFAD mice that overexpress the human APP gene with the Swedish (K670N, M671L), Florida (I716V), and London (V7171) mutations, and the PS1 gene with the M146L and L286V FAD mutations, or in APPSwe/PS1dE9 mice splenomegaly was not documented (Manna et al., 2021; Unger et al., 2020). Likewise, in the present study, we did not observe spleen enlargement in Tg-SwDI transgenic mice, which are widely used for AD and CAA basic and preclinical research. It has been suggested that chronic inflammatory and autoimmune processes can trigger spleen enlargement and pathological changes (Kapadia et al., 2018; Ma et al., 2022; Marchese et al., 2014; Zhang et al., 2020). Thus, Zhang and collaborators demonstrated that peripheral LPS administration caused systemic inflammation and splenomegaly in C57BL/6 mice (Zhang et al., 2020). However, results in other strains with documented inflammatory response but normal spleen size and morphology do not support this explanation as the sole cause of the splenomegaly observed in 3xTg-AD mice. On the other hand, the three mentioned above APP-Tg mouse strains, 5xFAD, APPSwe/PS1dE9, and Tg-SwDI, do not contain human tau gene with the P301L mutation present in 3xTg-AD mice (Oddo et al., 2003), and whether or not the presence of human tau in 3xTg-AD brain plays a role in the spleen enlargement is still being discussed.

In humans, splenomegaly has been documented in patients with cancer, autoimmune and inflammatory diseases, viral, bacterial, or parasite infections, liver pathologies, or some hereditary metabolic disorders (Pozo et al., 2009). It is not yet clear whether the splenomegaly observed in 3xTg-AD mice is related to AD-like pathology and whether our results, together with those reported previously, can help clarify the mechanism of brain-spleen axis involvement in AD or aid in the development of new therapeutic and diagnostic tools.

We also detected ThioS-positive amyloid in the spleens of 3xTg-AD but not Tg-SwDI mice. Previously, Manna and collaborators documented Congo-red amyloid deposits in the spleens of 18–24-month-old 3xTg-AD and 5xFAD mice, although, in 5xFAD animals, splenomegaly was not observed (Manna et al., 2021). Congo-red and ThioS bind to beta-sheet-enriched fibrils of various proteins, and specific antibodies should be used to determine which amyloid is accumulated in the spleen of 3xTg-AD mice. In this study, we showed for the first time that ThioS-positive amyloid aggregates in the spleen are specifically recognized by anti-mouse IgG antibodies, pointing to immunoglobulin-associated amyloidosis. Interestingly, St-Amour and collaborators documented a 5.4-fold increase in IgG concentration in the serum and a trend toward an increase in IgG-secreting cells in the spleen in 3xTg-AD mice (St-Amour et al., 2019). Whether the latter can explain our findings requires further investigation. In humans, immunoglobulin light-chain amyloidosis (AL) has been shown to comprise a major subset of the spleen amyloid cases (Chiu et al., 2023). Interestingly, the spleen with AL-type amyloidosis was frequently normal in size or only mildly enlarged (Chiu et al., 2023), like findings seen in 5xFAD mice; however, in the latter case, the authors did not investigate which amyloid composes CR-positive aggregates (Manna et al., 2021).

Likewise, we demonstrated for the first time that the spleen amyloid aggregates in 3xTg-AD mice do not bind to anti-Aβ specific antibodies, thus ruling out the hypothesis that the peripheral clearance of Aβ by the spleen is the cause of spleen amyloidosis (Yu et al., 2022). Interestingly, while 5xFAD mice do exhibit spleen amyloidosis without signs of splenomegaly, Tg-SwDI mice do not present any pathology in the spleen. In contrast, 3xTg-AD mice manifest both pathologies: splenomegaly and spleen amyloidosis. Many questions remain unanswered, and further experiments using different APPtg models will likely be required to address them.

It has been reported previously that the percentage of neutrophils in the spleen increases, in both humans and mice, in a variety of pathologies such as cancer, autoimmune diseases, inflammation, infection, and stroke (Kolaczkowska & Kubes, 2013; Zahng et al., 2020; Freire-Antunes et al., 2023; Liao et al., 2024; Guo et al., 2025). The authors suggested that this increase may be attributed to an augmented neutrophil infiltration into the spleen or splenic emergency granulopoiesis aimed at maintaining the immune balance during inflammation (Guo et al., 2025; Liao et al., 2024). Our results are consistent with a previous report on vast emergence of neutrophils in the spleen in 3xTg-AD (Manna et al., 2021), although in the latter study, the quantitative analysis was not performed. No increase in the percentage of neutrophils was observed in the spleen of Tg-SwDI mice, which highlights another distinctive feature of the 3xTg-AD mice.

The increase in the number of macrophages in the liver (resident Kupffer cells and infiltrating monocyte-derived macrophages) has been previously observed in several pathological conditions, such as inflammation, infection, tumor, or liver injury (Wen et al., 2021). Likewise, aging has been shown to be associated with an increased number of liver macrophages in rats (Bloomer et al., 2020). In the present study, we documented a significant increase in F4/80-positive cells in 15–16-month-old male 3xTg-AD mice (Fig. 4B) but not in female 3xTg-AD nor old Tg-SwDI or C57BL/6 mice of both sexes. Recently, Barber and collaborators reported elevated levels of F4/80-positive cells in the liver of male 3xTg-AD mice beginning at 12 months of age (Barber et al., 2024). Our results cannot be explained by inflammatory conditions or aging, as mentioned above, nor by the peripheral clearance of Aβ by the liver, because these features are characteristic also for old female 3xTg-AD mice and old male and female Tg-SwDI mice. Moreover, neither our colony male 3xTg-AD mice nor male 3xTg-AD mice in Barber´s lab exhibit plaque pathology, thus challenging the role of brain amyloid accumulation in changes in other tissues outside the central nervous system, such as the spleen and liver. Notwithstanding, it is worth mentioning a couple of interesting recent studies reporting the crosstalk between the liver and brain in two mouse models of AD: 5xFAD transgenic mice and C57BL/6 mice administered amyloid-beta peptide via intracerebroventricular (ICV) injection (Nitzan et al., 2019; Sweetat et al., 2023). Inflammatory cytokine IL-6 expression leading to impairment of mitochondrial functions in hepatic cells after intracerebral amyloid injection was documented; likewise, increased mitochondrial enzymatic activity in the brain and liver after intravenous mitochondrial transfer in these models was reported, although injected mitochondria were detected in the liver and not in the brain (Nitzan et al., 2019; Sweetat et al., 2023).

In conclusion, in this study, we documented striking differences in the periphery in two frequently used, well-established APP transgenic mouse models of AD. The key strength of 3 × Tg-AD mice is the development of neurofibrillary tangles, which are absent in the majority of other strains. This important feature makes them a desirable animal model for studying the interactions between tau and Aβ and evaluating therapeutic strategies directed against both proteins. However, the absence of Aβ aggregates in old 3xTg-AD male mice, on the one hand, and lack of the described pathologies, such as splenomegaly, spleen amyloidosis, an increase in neutrophils percentage in the spleen and macrophage percentage in the liver, in age-matched Tg-SwDI mice, another commonly used AD/CAA model, and in other APP transgenic mice reported previously, on the other hand, suggest taking into account the results observed in 3xTg-AD mice with caution to prevent erroneous interpretations due to artifacts.

## Supplementary Information

Below is the link to the electronic supplementary material.

Supplementary file1 (PDF 3030 KB)

## Data Availability

No datasets were generated or analyzed during the current study.
