# Supplementary material for: Splenomegaly, Spleen Amyloidosis and Neutrophil Infiltration are Present in 3xTg-AD, but not Tg-SwDI Mice
Source: Neuromolecular Med. 2025 Sep 4;27(1):61. doi: 10.1007/s12017-025-08884-8 (PMC12411599; doi:10.1007/s12017-025-08884-8)

Supplementary Information. Splenomegaly, spleen amyloidosis and neutrophil infiltration are present in 3xTg-AD, but not Tg-SwDI mice. Gonzalo Acero, Adrian Rodriguez-Lopez, Georgina Díaz, Daniel Esteban, Mónica Herrera-Ángeles and Goar Gevorkian*. Instituto de Investigaciones Biomédicas, Universidad Nacional Autónoma de México (UNAM); gokar@unam.mx.

Flow cytometry data for spleen and liver cells. Raw data for three mice from each group are shown (the total number of animals used in each group are mentioned in the manuscript).

A. Spleen cells

- C57BL/6: 3-month-old Male

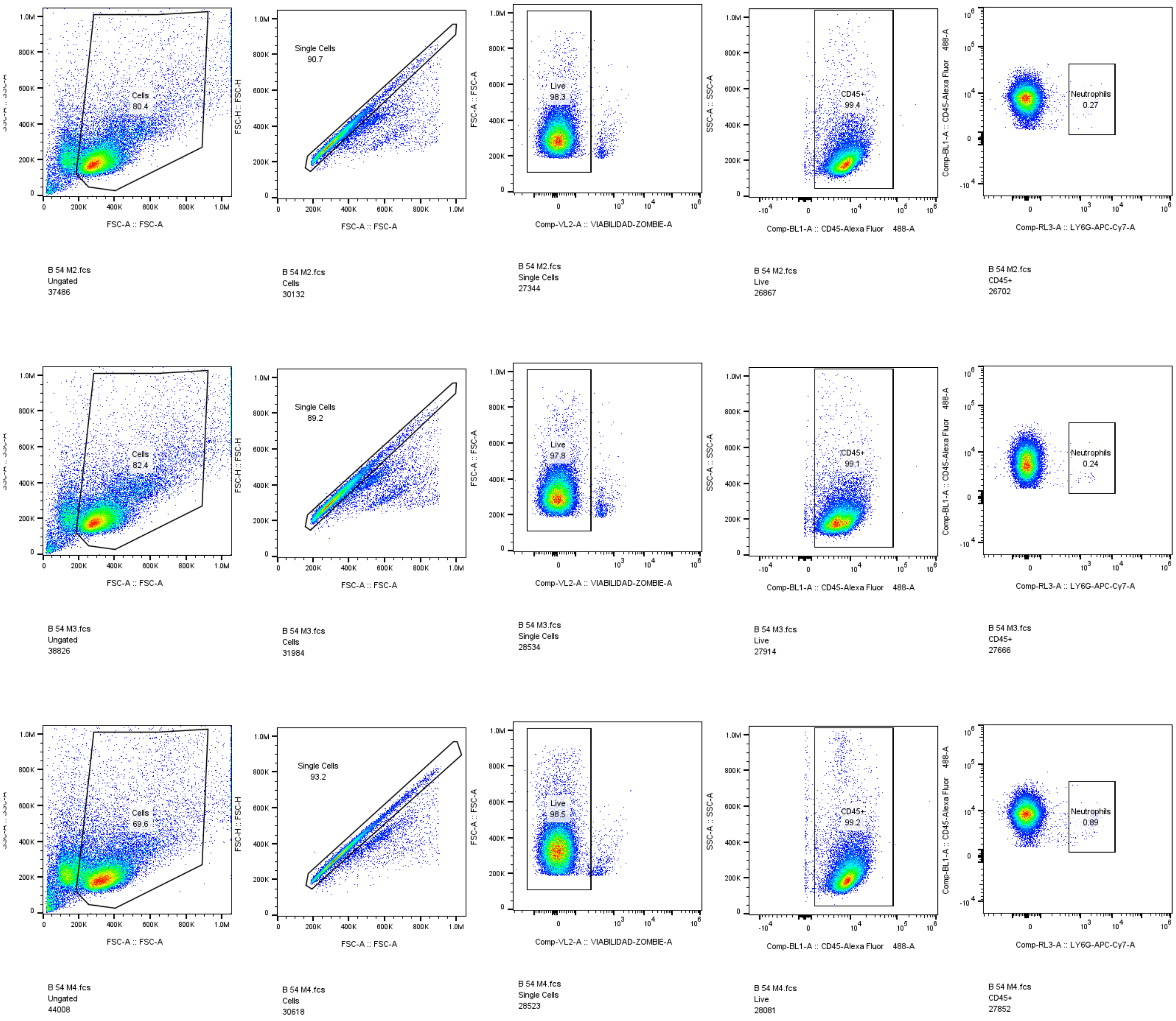

- C57BL/6: 3-month-old Female

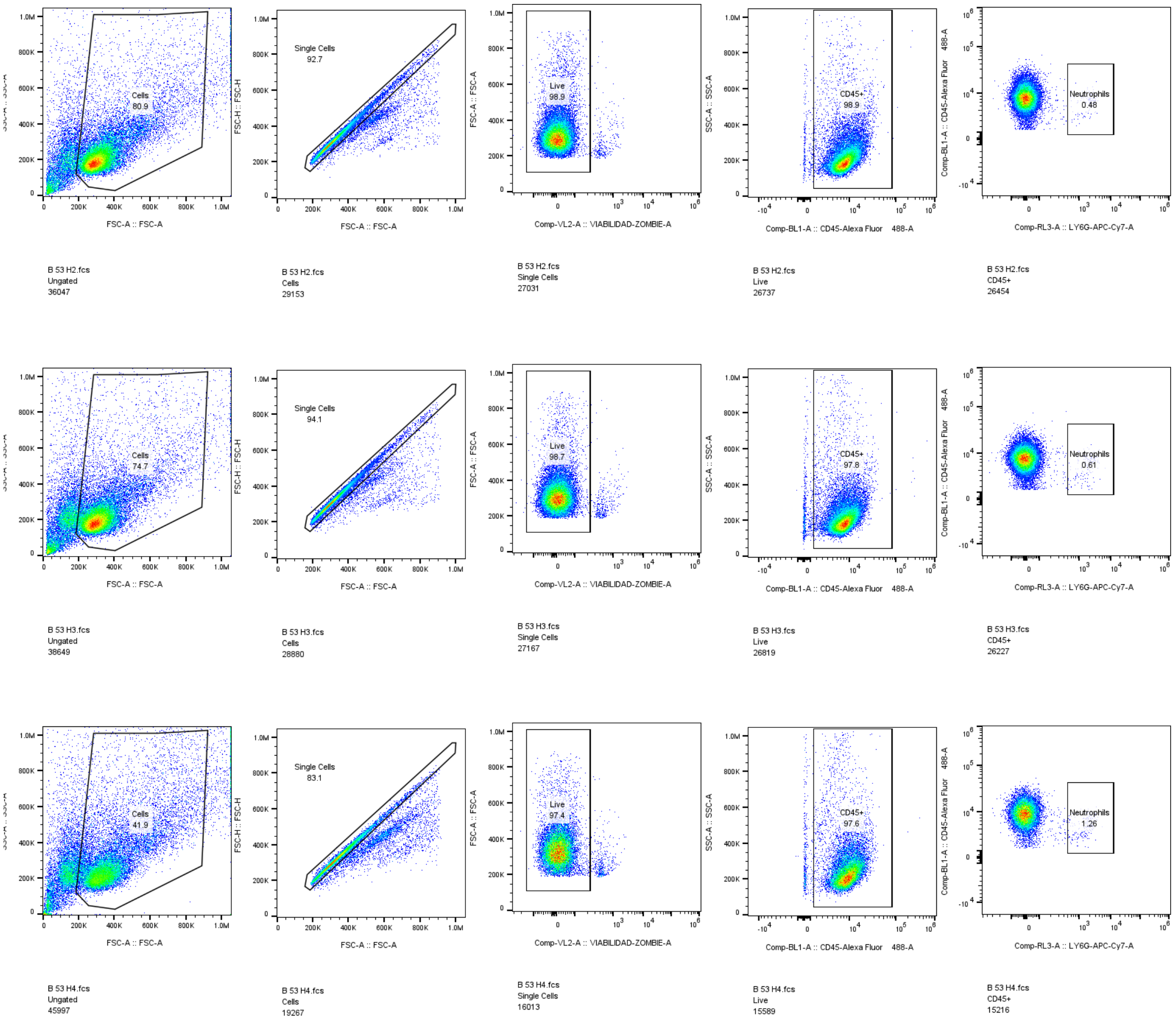

- 3xTg-AD: 3-month-old Male

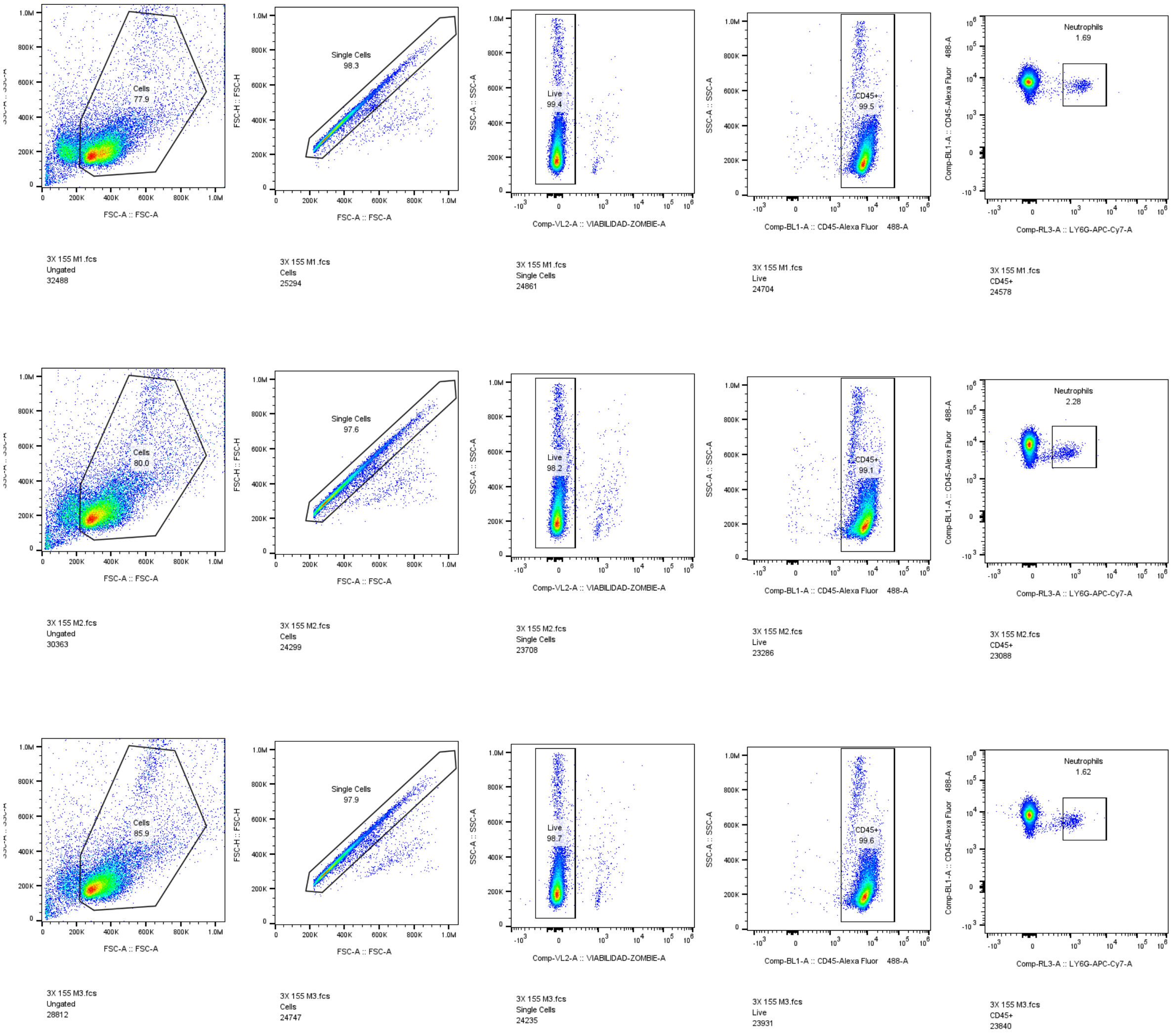

- 3xTg-AD: 3-month-old Female

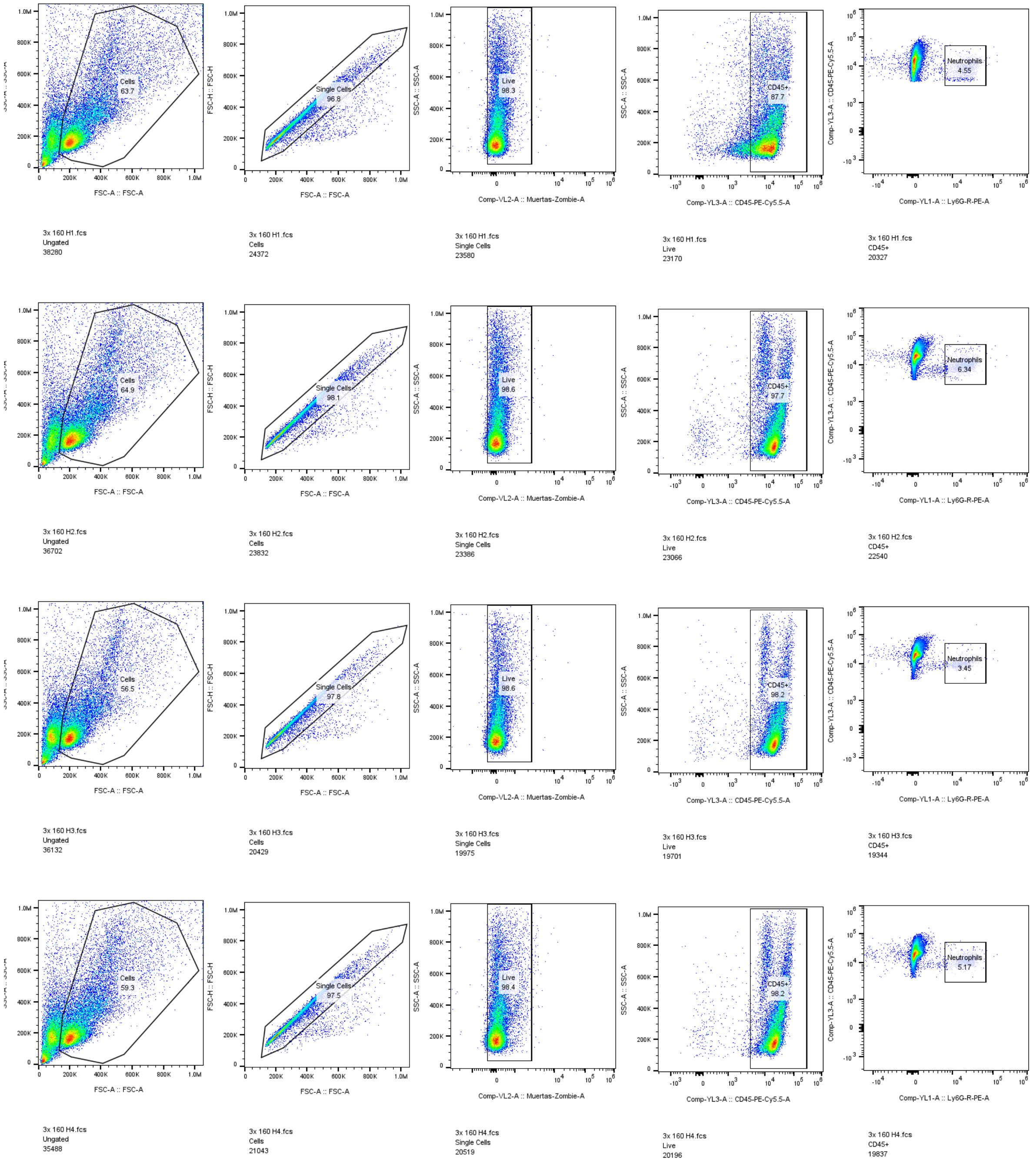

- C57BL/6: 15-16-month-old Male

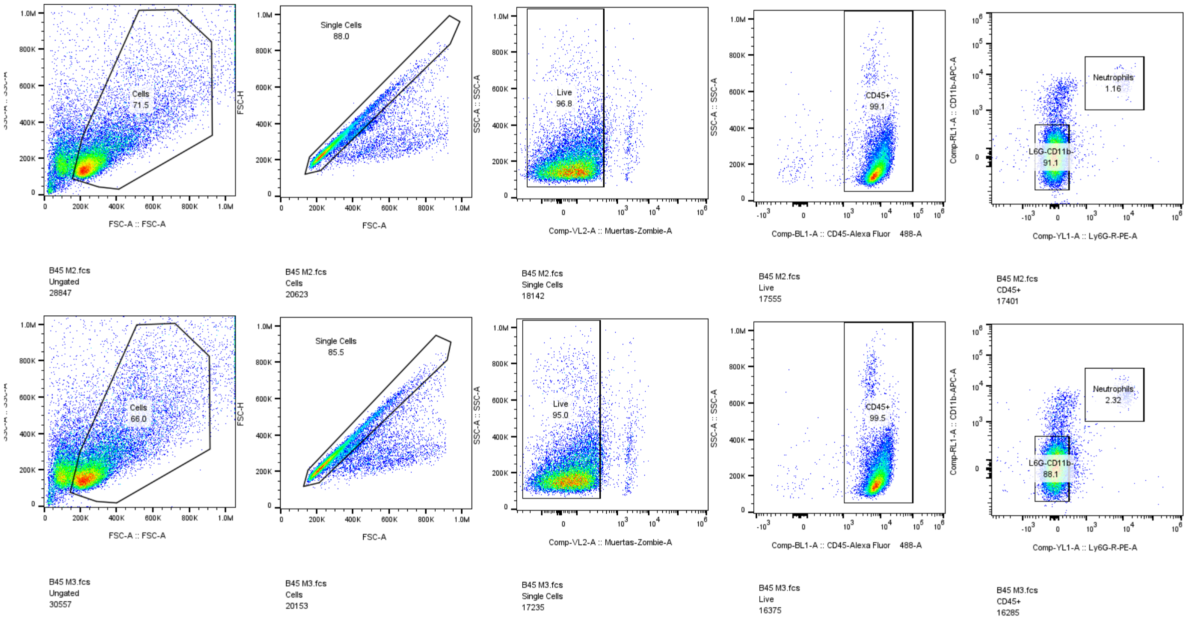

- C57BL/6: 15-16-month-old Female

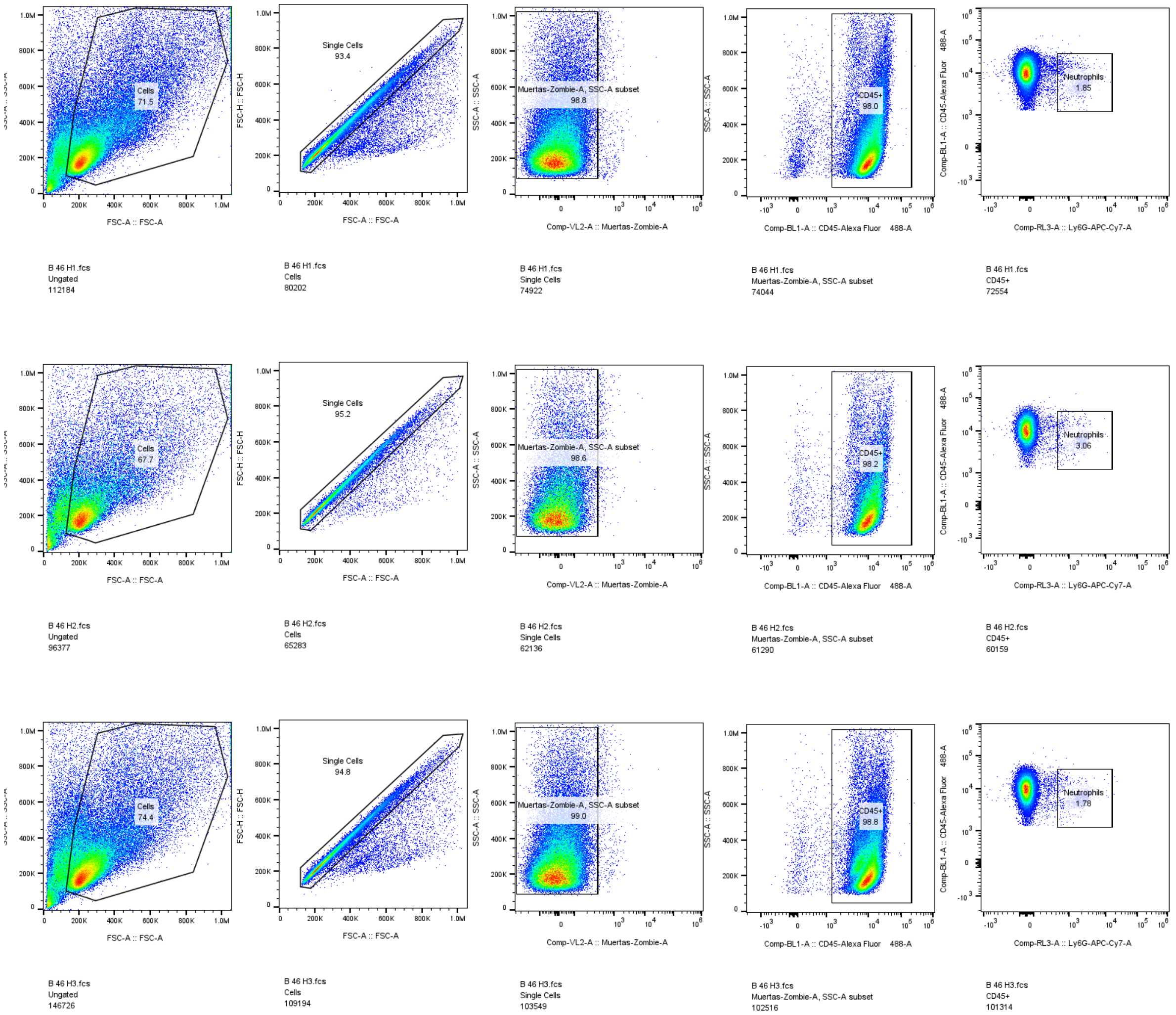

- 3xTg-AD: 15-16-month-old Male

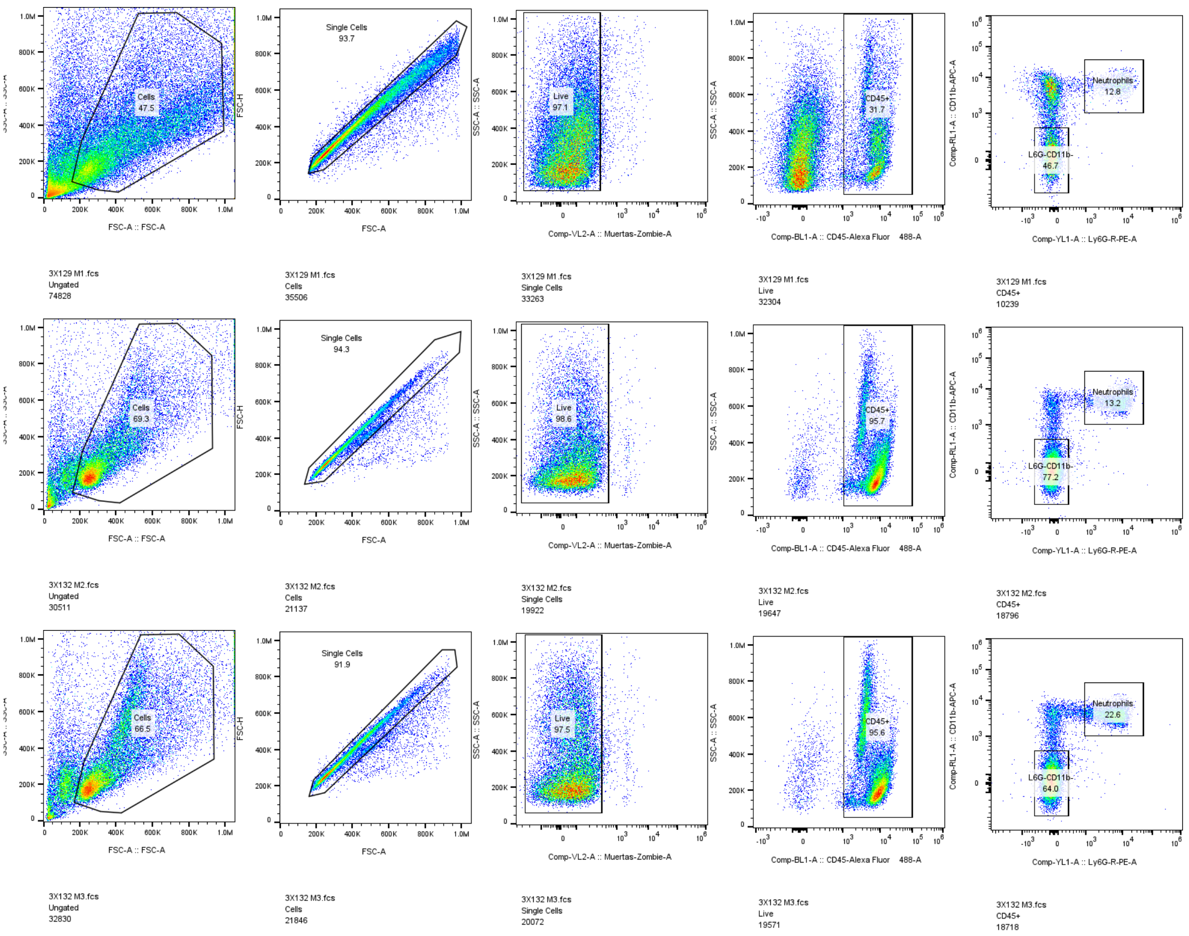

- 3xTg-AD: 15-16-month-old Female

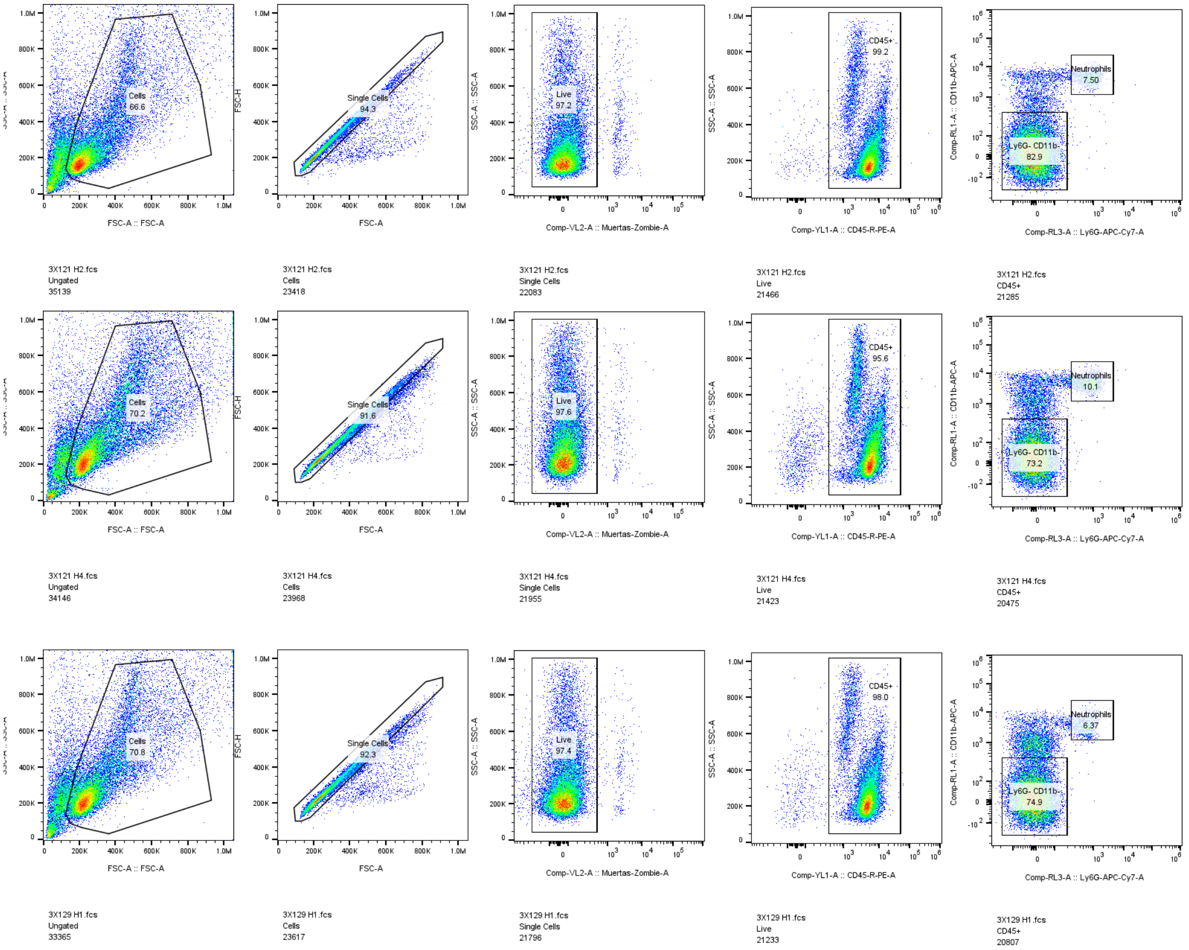

B. Liver Cells

- C57BL/6: 3-month-old Male

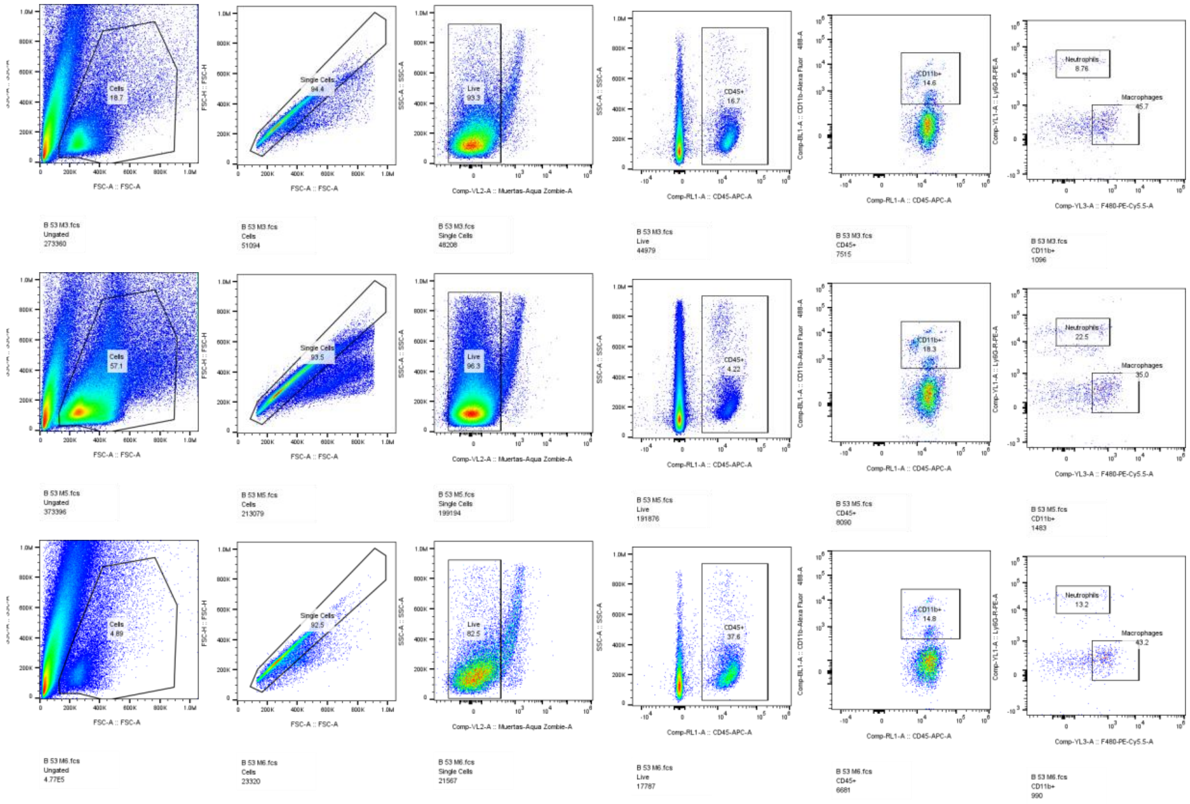

- 3xTg-AD: 3-month-old Male

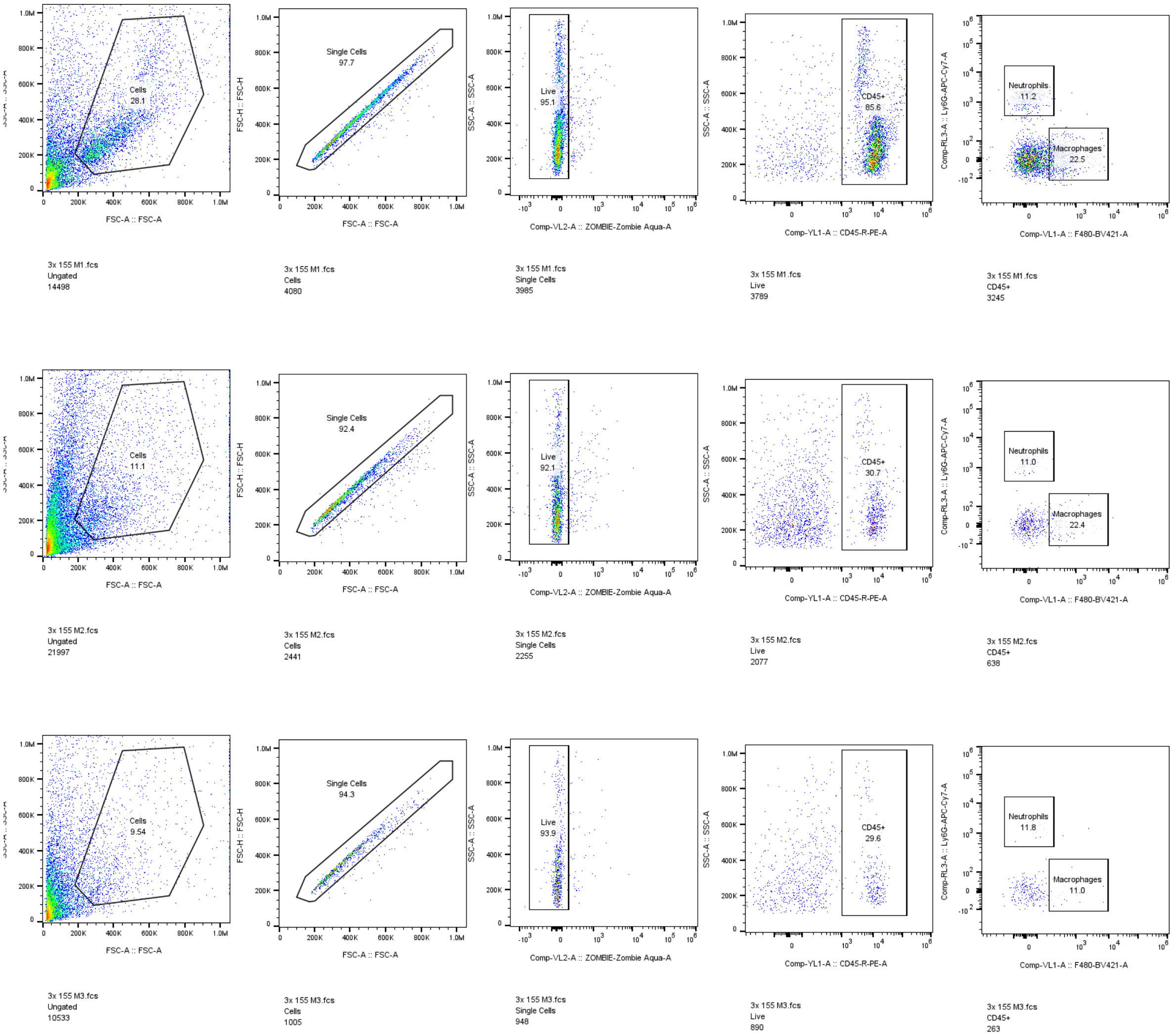

- C57BL/6: 15/16-month-old Male

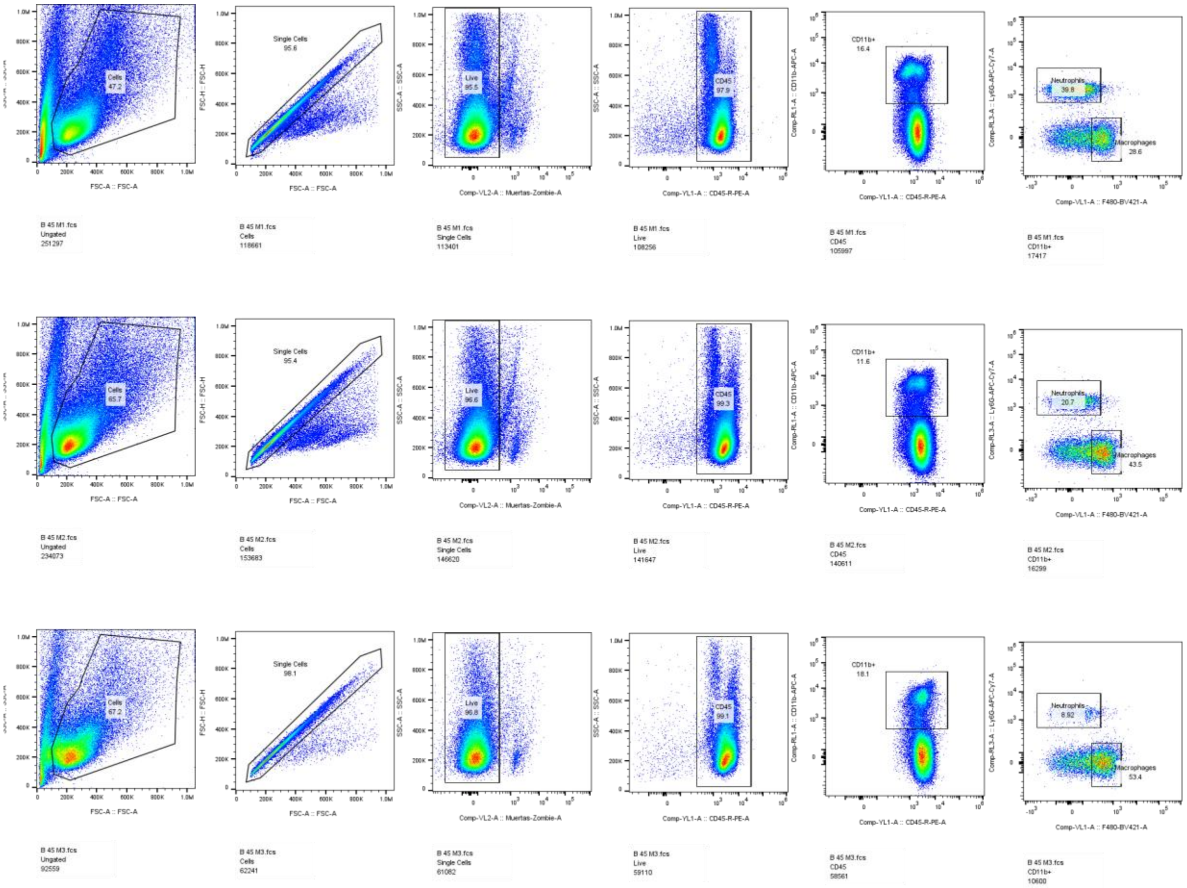

- 3xTg-AD: 15-16-month-old Male

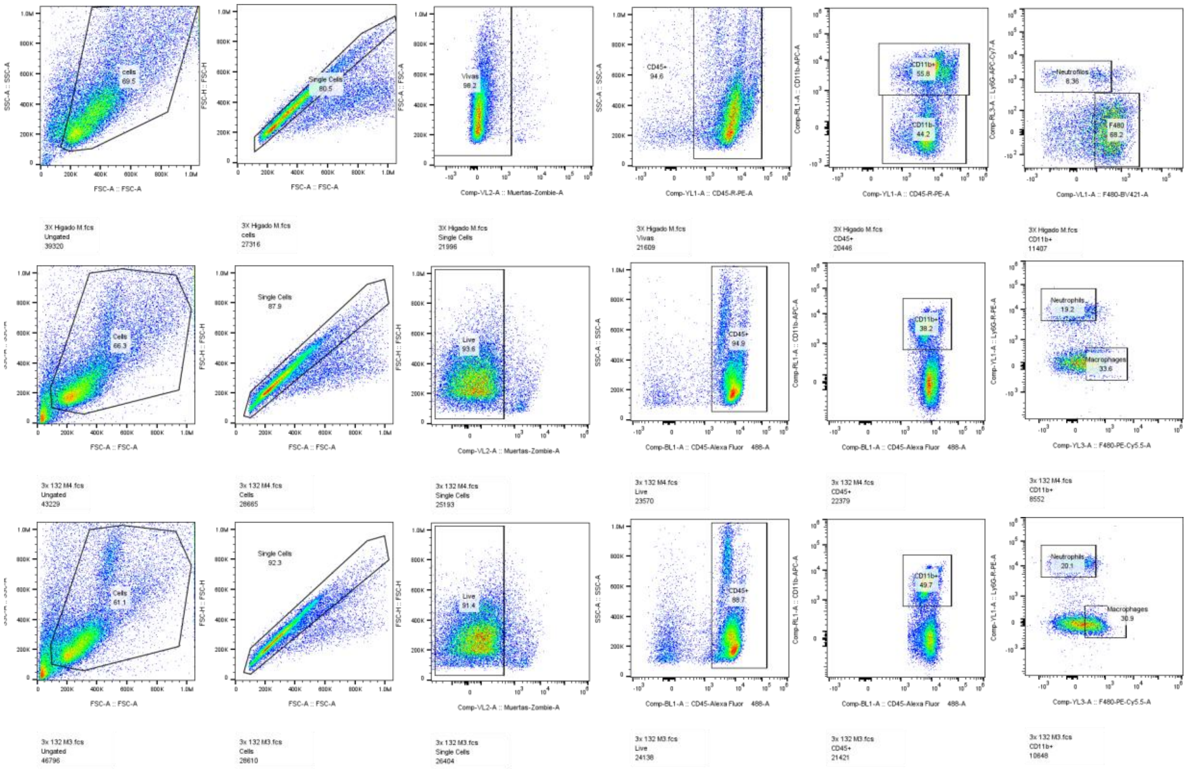

Supplement: Supplementary file 1 — Supplementary file1 (PDF 3030 KB) [file 12017_2025_8884_MOESM1_ESM.pdf]
